# Challenges to Achieving Measles Elimination, Georgia, 2013–2018

**DOI:** 10.3201/eid2611.200259

**Published:** 2020-11

**Authors:** Nino Khetsuriani, Ketevan Sanadze, Rusudan Chlikadze, Nazibrola Chitadze, Tamar Dolakidze, Tamta Komakhidze, Lia Jabidze, Shahin Huseynov, Myriam Ben Mamou, Claude Muller, Khatuna Zakhashvili, Judith M. Hübschen

**Affiliations:** Centers for Disease Control and Prevention, Atlanta, Georgia, USA (N. Khetsuriani);; CDC South Caucasus Office, Tbilisi, Georgia (N. Khetsuriani);; National Center for Disease Control and Public Health, Tbilisi (K. Sanadze, R. Chlikadze, N. Chitadze, T. Dolakidze, T. Komakhidze, L. Jabidze, K. Zakhashvili);; South Caucasus Field Epidemiology and Laboratory Training Program, Tbilisi (T. Komakhidze);; World Health Organization European Regional Office, Copenhagen, Denmark (S. Huseynov, M. Ben Mamou);; World Health Organization European Regional Reference Laboratory for Measles and Rubella, Luxembourg Institute of Health, Esch-sur-Alzette, Luxembourg (C. Muller, J.M. Hübschen)

**Keywords:** measles, measles elimination, measles epidemiology, measles virus, measles virus genetics, Georgia, European Region, vaccine-preventable diseases, viruses

## Abstract

Controlling measles outbreaks in the country of Georgia and throughout Europe is crucial for achieving the measles elimination goal for the World Health Organization’s European Region. However, large-scale measles outbreaks occurred in Georgia during 2013–2015 and 2017–2018. The epidemiology of these outbreaks indicates widespread circulation and genetic diversity of measles viruses and reveals persistent gaps in population immunity across a wide age range that have not been sufficiently addressed thus far. Historic problems and recent challenges with the immunization program contributed to outbreaks. Addressing population susceptibility across all age groups is needed urgently. However, conducting large-scale mass immunization campaigns under the current health system is not feasible, so more selective response strategies are being implemented. Lessons from the measles outbreaks in Georgia could be useful for other countries that have immunization programs facing challenges related to health-system transitions and the presence of age cohorts with historically low immunization coverage.

The country of Georgia, along with the other member states of the European Region (EUR) of the World Health Organization (WHO), is committed to achieving the goal of eliminating measles and rubella (*1*,*2*). However, the resurgence of measles in EUR since 2018 resulted in record-high numbers of cases and reestablished endemic transmission in some countries that had previously eliminated measles (*3*,*4*). Georgia is among the 12 EUR countries that have endemic transmission of measles and continues to experience periodic outbreaks (*4*,*5*).

Routine childhood immunization against measles was introduced in Georgia in 1966, resulting in reduction of incidence (Figure 1) (*5*,*6*). However, the excessive expansion of the list of contraindications to vaccination in the Soviet Union during the 1980s resulted in substantial immunity gaps (*7*,*8*). The immunization program deteriorated dramatically in the 1990s, during the first years after Georgia regained independence, but improved in the 2000s. Combined measles-mumps-rubella (MMR) vaccine (recommended at 12 months and 5 years of age) was successfully introduced in 2004. However, the accumulation of susceptible persons in cohorts born during the mid-1980s through the 1990s led to a series of measles outbreaks. A large-scale outbreak during 2004–2005 affected a wide age range, including older children and young adults (*5*). A nationwide measles-rubella supplementary immunization activity (SIA) in 2008, targeting the population 6–27 years of age, achieved only 50% coverage because of unjustified vaccine safety concerns (*9*). Another large-scale outbreak of measles occurred during 2013–2015 and was followed by the outbreak that began in 2017. Here, we review the status of measles in Georgia during 2013–2018, highlight challenges to achieving the elimination goal, and discuss approaches to address these problems.

**Figure 1:**
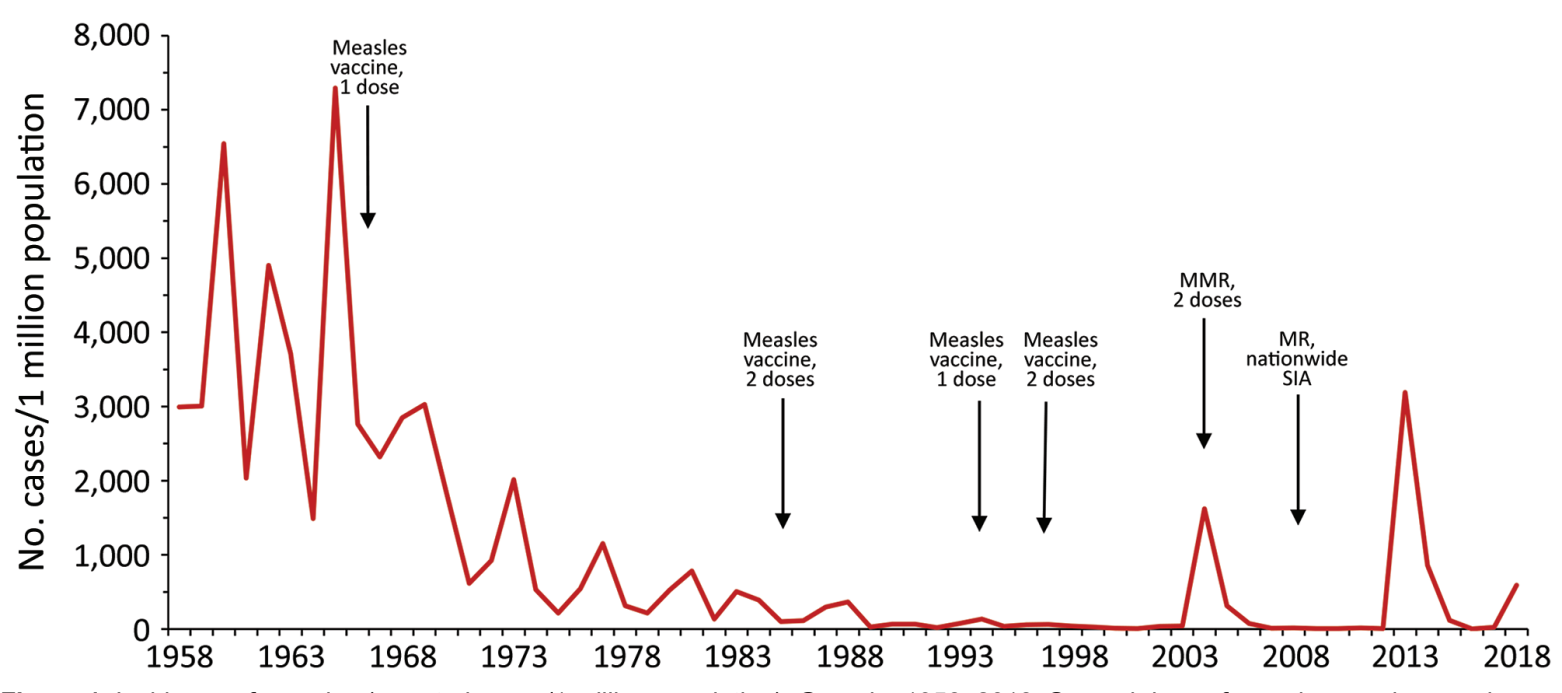
Incidence of measles (reported cases/1 million population), Georgia, 1958–2018. Second dose of measles vaccine was in the national immunization schedule during 1985–1993 but only 1982, 1983, and 1987 birth cohorts were vaccinated because of a lack of vaccine; the second dose was reintroduced in 1997 (*5*). MMR, measles-mumps-rubella vaccine; MR, measles-rubella vaccine; SIA, supplementary immunization activity.

## Methods

We reviewed measles surveillance data from the Georgia national surveillance system. National guidelines for measles surveillance, revised in 2017 (*10*; Appendix), follow WHO regional recommendations. Healthcare providers report suspected measles cases to district public health centers, which report the cases to the national Electronic Infectious Disease Surveillance System and conduct case investigation and response. The National Center for Disease Control and Public Health (NCDC) is responsible for the national level analysis and provides overall guidance. Case-based data on suspected measles cases are reported electronically each month to EUR. Laboratory testing is conducted by the National Measles and Rubella Laboratory at NCDC or, in rare cases, by private laboratories. Virus characterization is performed at the Regional Reference Laboratory in Luxemburg Institute of Health and at the National Measles and Rubella Laboratory. Measles virus sequences are reported to WHO through the Measles Nucleotide Surveillance (MeaNS) database (*11*,*12*).

We reviewed basic epidemiologic data for cases reported during 2013–2018 and conducted a detailed analysis of cases reported during 2013–2014, including descriptive epidemiology, occupational status, patterns of transmission, and costs to the public health system. An analysis of measles transmission across age groups was performed for a subset of cases for which the age group of the source (adult vs. child) could be determined from the Electronic Infectious Disease Surveillance System. We obtained information on expenses associated with outbreak response (costs of vaccine and personnel) from NCDC and population data from Georgia’s National Statistics Agency.

We obtained information on administrative MMR vaccine coverage from NCDC, supplemented by independent estimates from a coverage survey that we conducted in 2015–2016 (*13*; Appendix). In this survey, we estimated immunization coverage (nationwide and in 3 largest cities [Tbilisi, Batumi, and Kutaisi]) for the first MMR vaccine dose (MMR1) and the second MMR vaccine dose (MMR2) among children age-eligible to receive routine vaccinations in 2014 (2009 and 2013 birth cohorts). We estimated both coverage at the time of the survey and timely coverage by standard ages (MMR1 by age 24 months and MMR2 by age 72 months). We obtained additional information on the state of the immunization program in Georgia from WHO and GAVI (https://www.gavi.org) assessment reports. Additional details on epidemiologic methods are given in the Appendix.

The activities described in this report were determined by CDC to represent nonresearch. Therefore, institutional review board review was not applicable.

## Results

### Measles Epidemiology, 2013–2015

#### Descriptive Epidemiology

**Figure 2:**
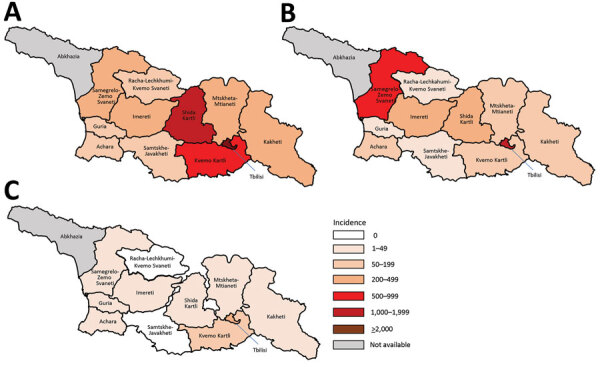
Incidence of measles (reported cases/1 million population), by region, Georgia, 2013 (A), 2014 (B), and 2015 (C). Rates for Abkhazia, currently outside government control, could not be calculated because of incomplete surveillance and lack of reliable population data.

A total of 11,495 measles cases were reported in Georgia during 2013–2015 (7,872 in 2013, 3,192 in 2014, and 431 in 2015) (Table 1; Figure 1; Appendix Figure), compared with 30 cases in 2012. The outbreak began in early 2013, and cases occurred predominantly among adults in Tbilisi, the capital city. The outbreak spread rapidly, affecting all regions by April and continued until mid-2015 (Figure 2). Tbilisi accounted for 47.0% of reported cases. The regions with the highest cumulative incidence per 1 million population during 2013–2015 were Shida Kartli (5,725) and Tbilisi (4,863), whereas Samtskhe-Javakheti (513) and Guria (763) had the lowest incidence.

**Table 1:** Epidemiologic characteristics of reported measles case-patients, Georgia, 2013–2018*

Characteristic	Reported measles cases, no. (%)		
	2013–2015 outbreak	2016	2017–2018 outbreak
Total cases	11,495 (100.0)	14 (100.0)	2,295 (100.0)
Final case classification category			
Laboratory-confirmed	1220 (10.6)	5 (35.7)	1,748 (76.1)
Epidemiologically linked	466 (4.1)	0 (0)	112 (4.9)
Clinically compatible	9,809 (85.3)	9 (64.3)	435 (19.0)
Sex			
M	6,000 (52.4)	8 (57.1)	1133 (49.4)
F	5,457 (47.6)	6 (42.9)	1162 (50.6)
Age group, y			
<1	1,130 (9.8)	4 (28.7)	229 (10.0)
1–4	1,707 (14.9)	6 (42.9)	321 (14.0)
5–9	962 (8.4)	0 (0)	167 (7.3)
10–14	751 (6.5)	1 (7.1)	174 (7.6)
15–19	1,286 (11.2)	0 (0)	164 (7.1)
20–24	1,707 (14.8)	1 (7.1)	302 (13.2)
25–29	1,750 (15.2)	1 (7.1)	304 (13.2)
30–39	1,546 (13.5)	0 (0)	441 (19.2)
40–49	477 (4.1)	0 (0)	143 (6.2)
>50	179 (1.6)	1 (7.1)	50 (2.2)
No. of doses of measles-containing vaccine received			
0	3,972 (34.6)	7 (50.0)	981 (42.7)
>1	1,346 (11.7)	4 (28.7)	183 (8.0)
1	1,020 (8.9)	4 (28.7)	123 (5.4)
2	326 (2.8)	0 (0)	60 (2.6)
Unknown	6,177 (53.7)	3 (21.3)	1131 (49.3)
Region			
Tbilisi	5,364 (47.0)	7 (50.0)	864 (37.6)
Achara	270 (2.4)	4 (28.7)	380 (16.6)
Guria	87 (0.8)	0 (0)	50 (2.2)
Imereti	742 (6.5)	0 (0)	469 (20.4)
Kakheti	610 (5.3)	1 (7.1)	36 (1.6)
Kvemo Kartli	1,159 (10.2)	0 (0)	87 (3.8)
Mtskheta-Mtianeti	286 (2.5)	1 (7.1)	29 (1.2)
Racha-Lechkhumi-Kvemo Svaneti	83 (0.7)	0 (0)	13 (0.6)
Samegrelo-Zemo-Svaneti	1,200 (10.5)	1 (7.1)	289 (12.6)
Samtskhe-Javakheti	83 (0.7)	0 (0)	28 (1.2)
Shida Kartli	1,509 (13.2)	0 (0)	36 (1.6)
Abkhazia	27 (0.2)	0 (0)	14 (0.6)

*Sex was not reported for 34 cases during 2013–2015. Region was not specified for 75 cases during 2013–2015. For Abkhazia, currently outside Georgia Government control, only cases treated in healthcare facilities in the government-controlled areas are reported.

**Figure 3:**
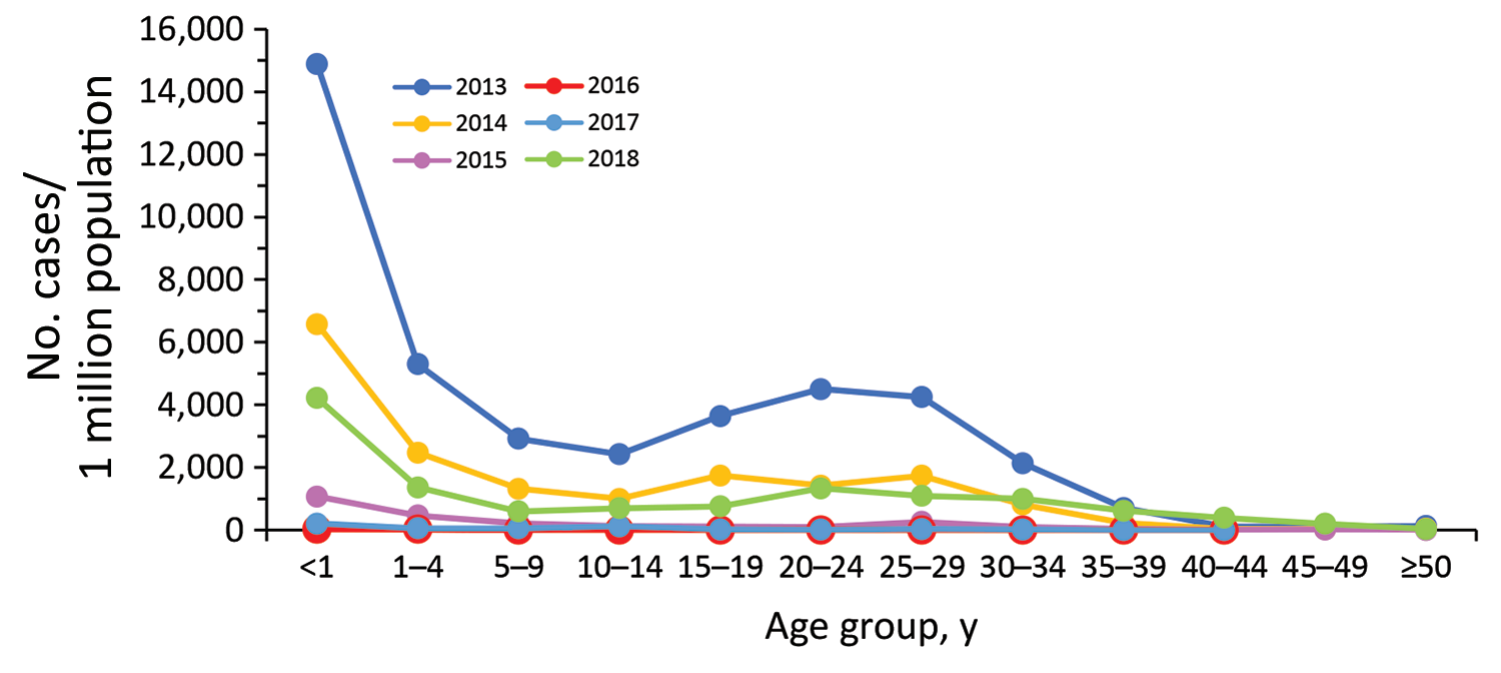
Incidence of measles (reported cases/1 million population), by age group and year, Georgia, 2013–2018.

**Figure 4:**
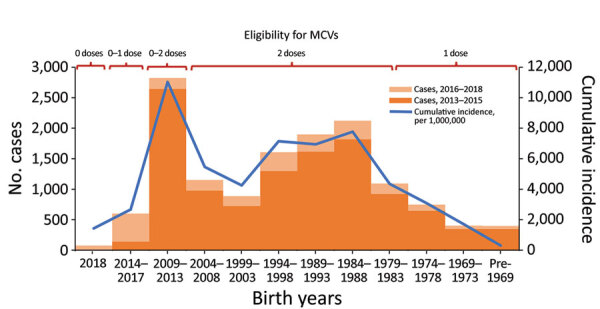
Reported measles cases during 2013–2015 and 2016–2018 and cumulative incidence (cases/1 million population) for 2013–2018, by birth year and eligibility to MCVs, Georgia. Children born during 2014–2017 gradually became eligible for the first dose of measles-mumps-rubella vaccine (MMR) by 2018 as the respective birth cohorts turned 1 year old. Children born during 2009–2013 were <5 years of age in 2013, at the start of 2013–2015 outbreak, and were either too young to be vaccinated (2013 cohort) or were eligible for the first dose of MMR vaccine only (2009–2012 cohorts), but gradually became eligible for the second dose of MMR vaccine by 2018, as the respective birth cohorts turned 5 years old. The 1981–2008 birth cohorts were eligible to 2 doses of MCV (measles vaccine, measles-rubella vaccine or MMR) through routine program, several supplementary immunization activities, or both. The 1959–1980 cohorts were eligible for 1 dose of measles vaccine through routine vaccination or catch-up immunizations conducted at the time of vaccine introduction. MCV, measles-containing vaccine.

**Figure 5:**
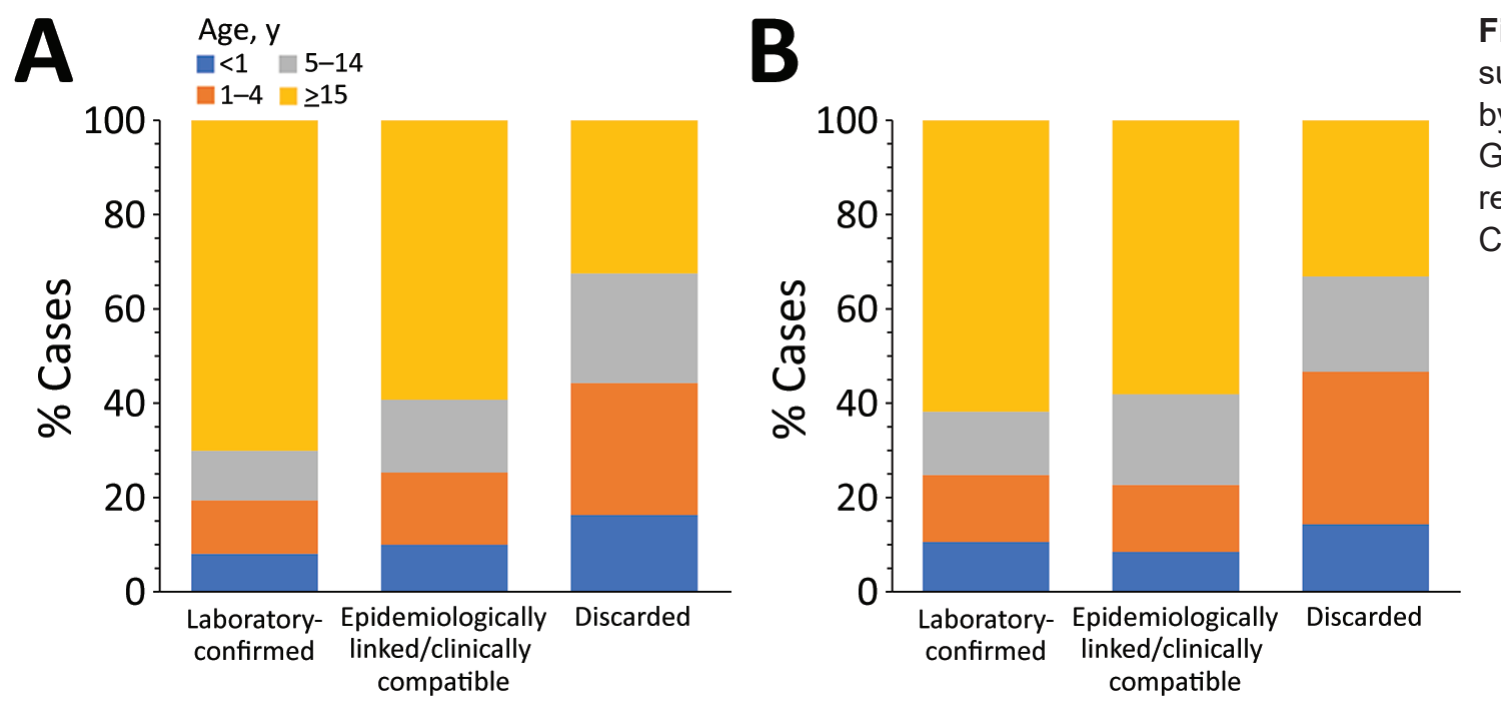
Age distribution of suspected measles case-patients, by final case classification category, Georgia, 2013–2018. A) Cases reported during 2013–2015. B) Cases reported during 2016–2018.

**Figure 7:**
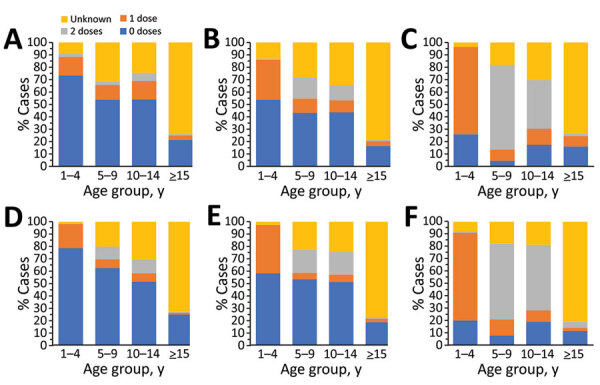
Vaccination status of suspected measles cases, by age group and final case classification category, Georgia, 2013–2015 and 2016–2018. A) Laboratory-confirmed cases reported during 2013–2015 (n = 1,220). B) Epidemiologically linked or clinically compatible cases reported during 2013–2015 (n = 10,275). C) Discarded cases during 2013–2015 (n = 289). D) Laboratory-confirmed cases reported during 2016–2018 (n = 1,753). E) Epidemiologically linked or clinically compatible cases reported during 2016–2018 (n = 556). F) Discarded cases during 2016–2018 (n = 608). Children <1 year of age, too young to be eligible for measles-mumps-rubella vaccination, are excluded.

Cases occurred across a wide age range (0–73 years; median 19 years), but most cases (60.4%) were among those >15 years of age (Table 1). The incidence was highest among children <1 year of age, followed by the 1–4-year- and 15–29-year age groups (Figures 3, 4). Almost 90% of the cases were in unvaccinated persons (34.6%) or those who had an unknown immunization status (53.7%); 8.9% had received 1 dose of measles-containing vaccine, and 2.8% had received 2 doses (Table 1). Distribution of cases by age group and immunization status by case-classification category are given in Figures 5–7.

Approximately one third (3,930 [34.3%]) of the 11,477 case-patients with hospitalization status reported were hospitalized. Hospitalizations were most common among unvaccinated persons (40.9% were hospitalized), followed by persons with unknown immunization status (33.5%), and were least common (18.6%) among recipients of >1 dose of measles-containing vaccine (p<0.001 by χ^2^ test). Complications were reported for 1,883 (16.4%) cases, most commonly pneumonia (1,328 cases [11.6%]) and diarrhea (587 cases [5.1%]). Encephalitis was reported in 9 (0.1%) cases. Adverse outcomes of pregnancy occurred in 5 cases (premature delivery in 3 cases and miscarriage in 2 cases). Four measles-related deaths occurred (case-fatality ratio 0.3/1,000 cases). Three of the fatal cases (in persons 11 months, 4 years, and 19 years of age) were in unvaccinated persons, and 1 was in a 36-year-old person with unknown immunization status.

#### Molecular Epidemiology

**Figure 8:**
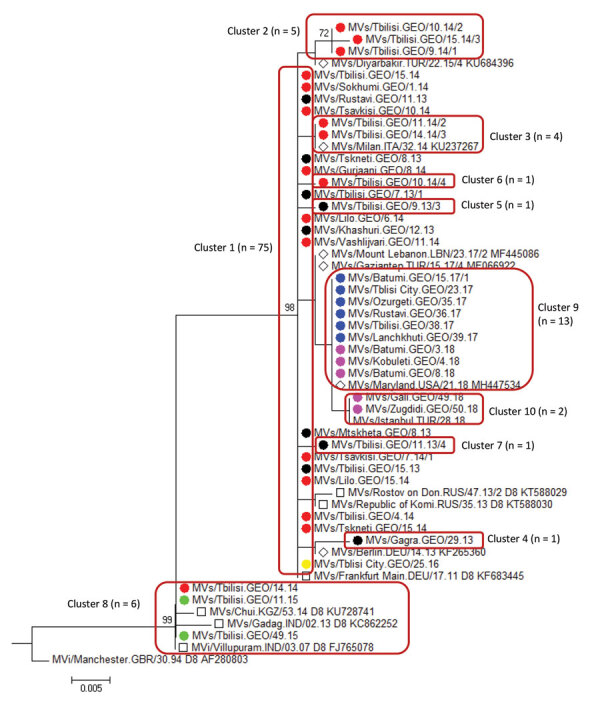
Genetic diversity of measles virus strains identified in Georgia, 2013–2018. Genotype D8 cluster of a phylogenetic tree is based on 450 nt of the measles virus nucleoprotein gene. The Kimura 2-parameter model and the neighbor-joining method in MEGA7 (*14*) were used, and only bootstrap values >70 are shown. The closest matches of the Georgia sequence variants identified by BLAST (https://blast.ncbi.nlm.nih.gov/Blast.cgi) are marked with a diamond; named strains of genotype D8 are marked with a square. For identical sequences, only the oldest and the most recent strains found in a certain location in a certain year are shown. The total number of sequences identified in each cluster are included in parentheses. Year of virus detection is indicated by colored circles: black for 2013, red for 2014, green for 2015, yellow for 2016, blue for 2017, pink for 2018. Scale bar indicates genetic distance, calculated based on the Kimura 2-parameter model, measured in nucleotide substitutions per site.

Molecular characterization of 93 measles viruses detected during 2013–2015, mostly in eastern Georgia, identified a single genotype (D8) with 9 different sequence variants (8 belonged to the Frankfurt-Main lineage, and 1 was identical to the Villupuram named strain) (Figure 8). The Frankfurt-Main variant (cluster 1) was the predominant strain associated with the outbreak (n = 74). This strain, first detected in Tbilisi in February 2013, became widespread during 2013–2014 but was not seen in 2015. Cluster 2 was represented by 5 strains from the Frankfurt-Main lineage (4 identical ones and 1 with 1 nucleotide difference) detected during February–April 2014. Another cluster of 4 sequences from March 2014 also differed from the Frankfurt-Main variant by 1 nucleotide (cluster 3). The July 2013 strain from Gagra (cluster 4) (in Abkhazia, currently outside Georgia government control) was clearly distinct from all other strains in the Frankfurt-Main lineage and most likely represents a separate introduction. Three other sequences, which differed from the Frankfurt-Main variant by 1 nucleotide each, were also identified (clusters 5–7). The lack of identical sequences from elsewhere in GenBank suggests that these strains could have evolved locally from the main Frankfurt-Main variant. Six sequences (1 from April 2014 and all 5 sequences from March–December 2015) were identical to the Villupuram variant (cluster 8), representing >1 separate introduction.

#### Virus Transmission across Age Groups

Among the 1,157 cases during 2013–2014 for which the age group of measles source was determined, the source of transmission in most cases (67.2%) was an adult (defined as >15 years of age) (Table 2), but the distribution of adult and child sources varied by the age of cases (p<0.001). Cases in adults were significantly more likely than those in children to have another adult as the source of infection (81.5% vs. 51.7%; odds ratio 4.0, 95% CI 3.0–5.2; p<0.001). Adult sources accounted for >50% of the cases among adults, infants <1 year of age, and older children (10–14 years of age), whereas young children (1–9 years of age) contracted measles primarily from other children (Table 2).

**Table 2:** Measles transmission sources for adult and child case-patients, by age group of measles case-patients, Georgia, 2013–2014*

Age of measles case-patients	Age group of measles source, no. (%)	
	Adult (>15 y)	Child (<15 y)
All ages, n = 1,157	778 (67.2)	379 (32.8)
Children <15 y, n = 545	282 (51.7)	263 (48.3)
<1 y, n = 151	118 (78.2)	33 (21.8)
<6 mo, n = 48	43 (89.6)	5 (10.4)
1–14 y, n = 394	164 (41.6)	230 (58.4)
1–4 y, n = 168	69 (41.1)	99 (58.9)
5–9 y, n = 112	35 (31.3)	77 (68.7)
10–14 y, n = 114	60 (52.6)	54 (47.4)
Adults >15 y, n = 612	496 (81.1)	116 (18.9)
15–19 y, n = 158	117 (74.1)	41 (25.9)
20–24 y, n = 145	128 (88.3)	17 (11.7)
25–29 y, n = 127	108 (85.0)	19 (15.0)
>30 y, n = 182	143 (78.6)	39 (21.4)

*Includes 1,157 cases reported during 2013–-2014 for which the age group of the measles transmission source (adults age >15 y vs. children age <15 y) could be established from the available surveillance data.

#### Population Groups Affected

Information on patient occupation was reported for 6,441 (58.2%) cases during 2013–2014. Almost half (48.1%) of them occurred among children not attending daycare (21.6%) or adults not working regularly outside the home (26.5%) (Table 3). Schoolchildren accounted for 17.5%, college students for 5.2%, and children attending daycare for 4.2% of the cases. Persons involved in direct customer service accounted for 7.5% of the cases. Healthcare facility (HCF) employees and medical or nursing students accounted for 3.9% of the cases, whereas 5.7% of the cases occurred among military or police.

**Table 3:** Occupations of reported measles case-patients, Georgia, 2013–2014

Occupation of case-patients	No. (%)
Adult not working outside the home	1,704 (26.5)
Unemployed	1,011 (15.7)	
Housewife	693 (10.8)
Child not attending daycare	1,392 (21.6)
School student (all grades)	1,127 (17.5)
Customer services (e.g., employees of banks, stores, casinos, restaurants)	484 (7.5)
Military or law enforcement	369 (5.7)
Military	239 (3.7)	
Law enforcement	130 (2.0)
College or vocational school student	334 (5.2)
Child attending daycare	271 (4.2)
Healthcare facility employee or medical student	250 (3.9)
Healthcare facility employee	209 (3.2)	
Medical student	41 (0.6)
Government or office worker	141 (2.2)
Other	369 (5.7)
Total with occupation information reported	6,441 (100)

Transmission associated with HCFs was observed in 123 cases linked to 30 different clusters, which also involved an additional 53 cases for which transmission occurred outside an HCF. The settings for HCF-associated transmission included major pediatric hospitals in Tbilisi, ambulance services, a cardiology clinic, infectious disease hospitals, a military hospital, and dental clinics.

#### Outbreak Response and Cost

The outbreak response activities and additional funds were mandated by the Prime Minister and the Minister of Health of Georgia. During 2013–2015, a total of 272,000 additional doses of MMR vaccine were procured. The immunization response included contact vaccination and offering MMR vaccine free of charge for all unvaccinated children <7 years of age, initially in Tbilisi, then nationwide. Subsequently, the eligible age group was expanded to those >30 years of age. Targeted special groups included healthcare workers and military personnel. Vaccine uptake was generally low (except among the military); 170,000 doses (62.5% of the available doses) were administered (85,000 doses to children 2–14 years of age, 41,000 doses to adults 15–29 years of age, 7,000 doses to healthcare workers, and 37,000 doses to contacts of measles case-patients and military personnel). Because of the substantial numbers of cases among the military, military personnel were considered potentially exposed or at high risk for exposure and vaccinated under the contacts category.

The total direct cost of additional vaccines and salaries for public health personnel during 2013–2015 was $720,000 (USD), of which $663,000 (92%), including $245,000 provided by the US government, was used for purchasing vaccines. The average direct cost per measles case for the public health system during this outbreak was $63.

### Measles Epidemiology, 2016–2018

Only 14 cases were reported in 2016 (Table 1), including a 3-case cluster in Tbilisi in June. The measles virus identified from that cluster was Frankfurt-Main, identical to the main outbreak strain circulating during 2013–2014 (cluster 1) (Figure 8). The 26-month interval since the last detection of this strain (in 2014) suggests a new introduction rather than continued transmission.

Ninety-six measles cases were reported in 2017 (Table 1), 92 (95.8%) of which occurred during August–December. The first 2 laboratory-confirmed cases occurred in April, 7 months after the previous laboratory-confirmed case. An outbreak of 16 cases during August–September began in Guria and was notable for its very high proportion of cases linked to HCF-associated transmission (13 cases [81.3%]), including 3 cases among healthcare workers. Measles activity further increased in late 2017, starting with school-based outbreaks in 2 districts of Achara and subsequently spreading to the regional capital Batumi. In 2017, Achara (65 cases) and neighboring region Guria (13 cases) accounted for 78 (81.3%) of cases in Georgia.

**Figure 9:**
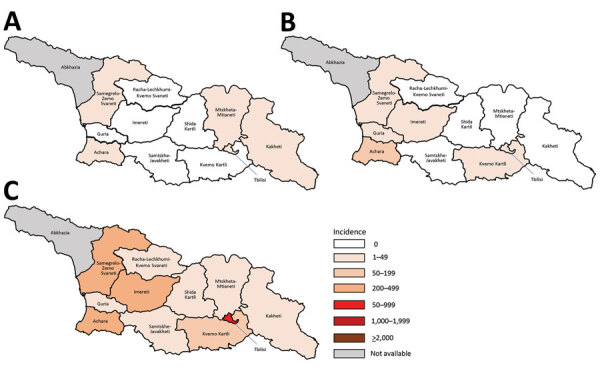
Incidence of measles (reported cases/1 million population), by region in Georgia, 2016 (A), 2017 (B), and 2018 (C). Rates for Abkhazia, currently outside government control, could not be calculated because of incomplete surveillance and lack of reliable population data.

The outbreak expanded in 2018, resulting in 2,199 reported cases (Table 1; Figures 1, 3, 9; Appendix Figure). During 2017–2018, the 4 regions with the highest cumulative incidence (cases/1 million population) , Achara (1,119), Imereti (911), Samegrelo-Zemo Svaneti (894), and Tbilisi (760), accounted for 2,002 (87.3%) cases. Two unvaccinated case-patients (ages 11 months and 16 years) died in 2018. The age distribution and immunization status of case-patients during 2017–2018 was comparable to the 2013–2015 period (Table 1; Figures 5–7). As seen during 2013–2015, most affected groups in 2016–2018 included birth cohorts too young to be vaccinated or age-eligible for MMR1 only, as well as young adults born during the 1980s and 1990s (Figure 4).

Measles virus sequences from 2017–2018 (n = 15) were detected across 8 regions and belonged to genotype D8. Thirteen identical strains (cluster 9) (Figure 8) detected during April 2017–February 2018 differed by 2 nucleotides from cluster 1, the predominant strain during 2013–2014. The other 2 identical sequences from December 2018 (cluster 10) were 1 nucleotide different from the rest of the 2018 strains and were identical to a virus identified earlier (July 2018) in Turkey (Figure 8).

Outbreak response activities included intensifying contact tracing and case-finding, enhancing surveillance and testing, reviewing immunization records of children in affected areas, and offering MMR vaccine free of charge to contacts and unvaccinated and undervaccinated persons <40 years of age. During 2017–2018, approximately 60,000 additional doses of vaccine were procured, and 47,000 doses were administered as part of the outbreak response. In November 2018, Georgia’s healthcare law was amended to make routine childhood immunizations mandatory (*15*). In early 2019, the policy of mandatory MMR vaccination for certain occupational groups, including healthcare workers, was introduced (*16*). The National Strategic Plan for Measles and Rubella Elimination was developed and is pending government approval.

### Immunization Coverage

**Figure 10:**
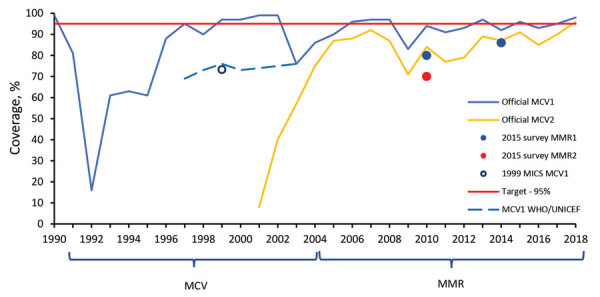
Immunization coverage with measles-containing vaccines in Georgia, 1990–2018. WHO/UNICEF estimates are included for 1997–2003, when official estimates were unreliable because of uncertainty in population numbers. WHO/UNICEF estimates are in agreement with the official estimates from 2003 to present. MCV, measles-containing vaccine; MCV1, first dose of MCV; MCV2, second dose of MCV; MICS, Multiple Indicator Cluster Survey; MMR, measles-mumps-rubella vaccine; MMR1, first dose of MMR; MMR2, second dose of MMR; WHO/UNICEF, World Health Organization/United Nations Children’s Fund.

The administrative coverage fluctuated over time and mostly remained below the national target (95%) (Figure 10). However, in 2015 and 2017, reported MMR1 coverage reached 95%–96% and MMR2 coverage reached 90%–91%. In 2018, coverage for both doses exceeded 95% for the first time (98% for MMR1 and 96% for MMR2).

**Figure 11:**
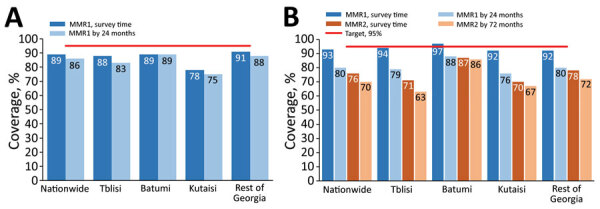
Coverage with the first and the second doses of measles-mumps-rubella vaccine, according to an immunization coverage survey, Georgia, 2015–2016. A) 2013 birth cohort. B). 2009 birth cohort. MMR, measles-mumps-rubella vaccine; MMR1, first dose of MMR; MMR2, second dose of MMR.

A coverage survey conducted during 2015–2016 demonstrated that by the time of the survey, 89% of children born in 2013 and 93% of children born in 2009 had received MMR1; and 76% of children born in 2009 had received MMR2 (Figure 11). Timely coverage was lower, particularly in the 2009 cohort, highlighting the problem with delayed vaccinations, although MMR1 coverage by age 24 months improved from 80% in the 2009 cohort to 86% in the 2013 cohort. Timely MMR2 coverage in the 2009 cohort was 70%. Geographic variations were particularly notable for the 2009 cohort, with substantially higher coverage in Batumi than in other sites. MMR1 coverage in the 2013 cohort was lowest in Kutaisi. MMR2 coverage was low in all sites except Batumi (Figure 11).

### Main Performance Indicators for Measles Surveillance

**Figure 6:**
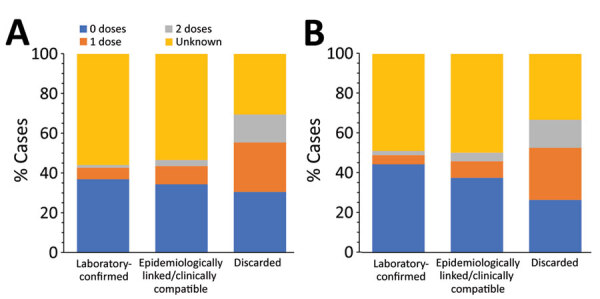
Vaccination status of suspected measles case-patients, by final case classification category, Georgia, 2013–2018. A) Cases reported during 2013–2015. B) Cases reported during 2016–2018.

During 2013–2017, the discarded case rates ranged from a high of 4.5/100,000 population in 2013 to a low of 1.2/100,000 population in 2016; the >2.0/100,000 population WHO target for this indicator was met only in 2013. Geographic variations were observed, with consistently low discarded case rates in some regions. In 2018, surveillance quality improved substantially, with a discarded case rate of 13.6/100,000 population nationwide and >2/100,000 population in all regions. The >80% target for timeliness of case investigation (*1*) was consistently met; during 2013–2018, case investigation was initiated within 48 hours of notification for >95% of suspected measles cases. The rate of laboratory investigation of cases (*1*) has improved substantially, from 13.3% during 2013–2015 to 79.6% in 2016 and 84.6% during 2017–2018, resulting in a decline in the proportion of clinically compatible cases among all measles cases from 85.3% during 2013–2015 to 19.0% during 2017–2018. Comparison of age distribution and vaccination status of suspected measles cases by final classification category indicated relatively minor differences between laboratory-confirmed cases and those classified as epidemiologically linked or clinically compatible; however, cases in all these categories differed substantially from discarded cases, which had lower proportions of adults and higher proportions of vaccinated persons (Figures 5,6,7). The highest proportions of unvaccinated cases were observed in the laboratory-confirmed category among children 1–4 years of age (who were age-eligible for MMR1 only), whereas the highest proportions of vaccinated children were observed among epidemiologically linked or clinically compatible cases in children 5–14 years of age (who were age-eligible for both MMR1 and MMR2) (Figure 7). In contrast, in the discarded category, most case-patients <15 years of age were vaccinated; 1-dose recipients were predominately children 1–4 years of age and 2-dose recipients children 5–14 years of age. The similarities between different categories of measles cases and their clear differences from discarded cases during large-scale outbreaks provide additional reassurance regarding the quality of measles surveillance in Georgia.

## Discussion

Measles epidemiology in Georgia during 2013–2018 shows widespread circulation and genetic diversity of measles viruses and points to persistent gaps in population immunity across a wide age range that have not been sufficiently addressed by interventions undertaken so far (*5*,*9*). Measles in Georgia is associated with substantial economic costs, disease, and deaths; its effects extend beyond the acute illness, as suggested by the recently demonstrated high risk for subacute sclerosing panencephalitis after measles outbreaks in Georgia (*17*).

Cases among children highlight challenges with routine immunization services. Previous suboptimal MMR1 coverage and vaccination delays, primarily because of unwarranted contraindications (*13*), likely contributed to the high incidence of measles among children. Although most children receive MMR vaccine, vaccination often happens years after the recommended ages, widening the window of susceptibility, particularly among those age-eligible to MMR1 only. 

High incidence among adults and <1-year-old infants results from continued susceptibility among persons born in the 1980s and 1990s (Figure 4) and is consistent with the results of a serosurvey conducted in Georgia immediately after the 2013–2015 outbreak (*18*), which demonstrated residual seronegativity to measles above the 7% susceptibility threshold needed for preventing outbreaks (*19*) among young adults. Seronegativity was 10.1% among persons 18–24 years of age, including 14.5% among college students and 8.0% among those 24–29 years of age (*18*). Analysis of measles transmission patterns demonstrated the important role of adults in virus circulation, suggesting that the adult population could potentially maintain measles transmission in Georgia. Along with widespread susceptibility among adults, small birth cohorts and the generally small number of children in households in Georgia (*20*) could have contributed to this finding.

Our findings highlight the urgent need to address population susceptibility across all age groups in Georgia. To improve immunity among children, ongoing catch-up immunization of unvaccinated and undervaccinated children should be accelerated, along with further strengthening routine immunization services. Educational efforts promoting awareness among parents and healthcare providers should be intensified to address needless delays attributable to unwarranted contraindications. Effective communication and stakeholder coordination will be needed to ensure the successful implementation of legislation endorsing mandatory childhood vaccinations in Georgia (*15*). Implementing mandatory MMR vaccination of certain occupational groups (*16*) and expanding this policy to include all college students could considerably reduce measles transmission among adults in high-risk settings, including HCFs, which have been a substantial contributor to outbreaks. However, reaching susceptible persons in the general adult population who account for a large proportion of cases, remains extremely challenging. The unsuccessful measles-rubella SIA conducted in 2008 (*9*) was a missed opportunity to close historic immunity gaps in Georgia. Conducting large-scale SIAs in Georgia’s present healthcare environment is not feasible because of the lack of defined catchment areas or populations, the voluntary nature of patient registration with HCFs, the lack of mechanisms or motivation for providers to identify and offer vaccinations to unregistered persons, and difficulties in locating historic records to ascertain vaccination status of adults. In addition, acceptance of mass immunizations among healthcare providers and public health professionals has been low since the SIA in 2008 (*9*). Under these circumstances, more selective and targeted efforts to control measles outbreaks are being implemented. The result of these efforts will depend primarily on the level of public acceptance. The suboptimal uptake of MMR vaccine among adults indicates the need for interventions to generate vaccine demand.

Information provided by the measles surveillance system is critical for guiding outbreak responses and documenting virus transmission. Measles surveillance in Georgia currently meets most performance indicators. Further improving the quality of case and outbreak investigations will help ensure that all chains of transmission are promptly identified and followed up.

Improved molecular surveillance, notwithstanding certain temporal and geographic gaps, helped demonstrate that virus introductions and local evolution likely contributed to continued transmission. At least 2 variants of measles virus (the main outbreak strain [cluster 1] and the strain in cluster 8) have likely established long-term (>12 months) transmission in Georgia during the 2013–2015 outbreak, but their circulation has been interrupted since then. Cluster 9, detected during April 2017–February 2018, possibly represents a new introduction. Given the slow rate of measles virus evolution (*21*) and a very low level of measles activity in 2016, the 2-nucleotide difference from the Frankfurt-Main strain probably would not have emerged over the 9-month period since its last detection in Georgia. Cluster 9 strains also might have circulated for >12 months, but no virus specimens were collected during March–November 2018 (the peak of the outbreak), preventing definitive conclusion.

Controlling measles outbreaks throughout EUR is crucial for achieving the regional elimination goal. The experience in Georgia demonstrates that without adequate and timely response, substantial susceptibility to measles can persist in settings with historically suboptimal coverage even after large-scale outbreaks, thus leaving room for future outbreaks. A similar pattern was observed in Ukraine and in Bosnia and Herzegovina, where, in the absence of appropriate response, the historically underimmunized birth cohorts were affected by repeated outbreaks of measles (*22*–*27*). In contrast, those countries in the former Soviet Union and Eastern Europe that successfully implemented wide-age SIAs, achieved elimination or substantial reduction of measles incidence for prolonged periods (*4*,*28*,*29*). However, implementing traditional SIAs is not feasible in many middle- and high-income countries of EUR. Lessons learned from Georgia could be useful for other countries with immunization systems facing similar challenges related to health-system transitions and the presence of age cohorts or population groups with historically low coverage.

Appendix Additional information about challenges to achieving measles elimination, Georgia, 2013–2018.
