# Supplementary material for: Challenges to Achieving Measles Elimination, Georgia, 2013–2018
Source: Emerg Infect Dis. 2020 Nov;26(11):2565–77. doi: 10.3201/eid2611.200259 (PMC7588556; doi:10.3201/eid2611.200259)
Supplement: Appendix — Additional information about challenges to achieving measles elimination, Georgia, 2013–2018. [file 20-0259-Techapp-s1.pdf]

Article DOI: https://doi.org/10.3201/eid2611.200259

Challenges to Achieving Measles Elimination, Georgia, 2013–2018

Appendix

Measles Epidemiology And Surveillance: Additional Details On Methods

According to Georgia's national guidelines for measles surveillance (*1*), which follows regional guidance (*2*) from the European Office of the World Health Organization, all suspected measles cases (i.e., cases which meet clinical case definition for measles) are notifiable within 24 hours. Following the review of clinical, epidemiologic and laboratory data, all suspected cases are classified into one of the following final classification categories: laboratory-confirmed, epidemiologically linked, clinically compatible, and discarded. Cases classified as laboratory-confirmed, epidemiologically linked and clinically compatible are included in the total count of reported measles cases. Discarded cases are excluded from measles case count, but they are still reported as a separate category for the purpose of monitoring measles surveillance quality.

Measles incidence rates were expressed as number of reported measles cases per 1 million population. Population data for calculating rates was obtained from the National Statistics Office of Georgia (*3*). Population data exclude regions currently not under Georgian government's control (South Ossetia and Autonomous Republic of Abkhazia). Georgia's total population in 2013 was 3.7 million, with the age distribution presented in Appendix Table 1. There were no substantial variations in the population size or age distribution during the period covered in this report. Region-specific rates were not calculated for Abkhazia because of incomplete surveillance (the only reported cases from Abkhazia were those treated at the healthcare facilities in regions under Georgian government control) and the lack of reliable population data. No data were available for South Ossetia.

Patterns for transmission of measles across age groups (adults versus children) was analyzed for cases reported in 2013–2014, for which the age group of the source of transmission could be determined from the Electronic Infectious Disease Surveillance System. Children were

defined as persons aged <15 years, adults, as persons aged ≥15 years. We analyzed the proportion of cases of different age groups by the age group of potential sources. X^2 test was used for statistical comparisons. In the analysis of the costs of measles outbreaks, the expenses were converted into USD using exchange rates at the time when they were incurred.

Various indicators are used to assess sensitivity, specificity, timeliness and completeness of measles surveillance (*1*,*2*). The main indicators reviewed in this report are included in the Appendix Table 2.

Immunization Coverage Survey: Methods

Below is the summary of the background and methods of the immunization coverage survey conducted in Georgia in 2015–2016 applicable to coverage with measles-mumps-rubella vaccine (MMR). The full report is available online (*4*).

Participating Institutions

U.S. Centers for Disease Control and Prevention (CDC), Center for Global Health, Global Immunization Division; CDC South Caucasus office, Field Epidemiology and Laboratory Training Program; National Center for Disease Control and Public Health (NCDC), Tbilisi, Georgia

Background

Vaccination against measles has been in place in Georgia since 1966. Since 2004, the national immunization schedule includes MMR vaccine at 12 months and 5 years.

Immunization coverage in Georgia had been high until 1990, but declined in the 1990s, during the immediate period after the regaining of independence and subsequent armed conflicts and economic crisis. Although immunization services have improved in the last decade, challenges remain, as demonstrated by continued occurrence of outbreaks of measles. As of 2015, at the time of planning the survey, national coverage estimates for the first and second doses of measles-containing vaccines (MCV1 and MCV2, respectively) reported by Georgia to WHO (Appendix Table 3) remained largely below the national target of 95% (*6*).

The accuracy of administrative coverage data was unclear because of difficulties with determining target populations, particularly in the cities where the continuous changes to health

care system had greatest impact on primary health care facilities (HCFs). The abolition of geographic catchment areas for HCFs, intense population movement, and existence of uncertain number of children not registered with HCFs resulted in greater difficulties with assessing coverage in large cities than in smaller towns and rural areas. Administrative coverage data have not been validated for over a decade, as no independent nationwide coverage surveys have been conducted in Georgia since a Multiple Indicator Cluster Survey (MICS) in 1999 (*8*). Immunization data could not be analyzed for the MICS survey conducted in 2005 because immunization cards for ~85% of households were not stored at home (*8*).

Because of the lack of independent validation of the coverage data in Georgia and ongoing uncertainty with target populations, we conducted a nationwide immunization coverage survey during 2015–2016 to assess coverage with vaccines included in the routine immunization schedule through 5 years of age.

Survey Design

Survey Population and Vaccine Doses Assessed

Most standard protocols for immunization coverage surveys (MICS, DHS, EPI cluster survey) only include vaccines given during the first year of life and first dose of measles vaccine, but this approach leaves out later doses, such as second dose of measles-containing vaccines, and doses after primary series for diphtheria-tetanus toxoids and polio vaccines. The coverage with vaccine doses recommended after 12 months of age in Georgia has not been independently assessed previously. Therefore, we decided to assess coverage with all vaccines included in the immunization schedule before the age 6 years.

Per NCDC request, and because of greater uncertainties with accuracy of reported coverage data in cities, the survey was designed to allow obtaining separate estimates for three largest cities of Georgia – Tbilisi (2015 population 1,100,000), Batumi (154,000), and Kutaisi (148,000), which together account for 38% of total population of the country (*3*). Therefore, these three cities and rest of Georgia were surveyed separately and nationwide estimates were obtained by pooling the data from these surveys. The areas currently not under Georgian Government control (South Ossetia and Autonomous Republic of Abkhazia) were excluded because of lack of population data, inaccessibility and security concerns.

We included in the survey children eligible for routine immunizations in 2014, the most recent year with available coverage data at the time of planning and initiation of the survey. These included three birth cohorts:

- Children born in 2014, eligible to receive vaccines recommended during the first year of life
- Children born in 2013, eligible to receive vaccines recommended during the second year of life, including first dose of MMR vaccine (MMR1), recommended at 12 months of age
- Children born in 2009, eligible to receive vaccines recommended during the sixth year of life, including second dose of MMR vaccine (MMR2), recommended at 5 years of age.

We estimated immunization coverage with age-appropriate vaccines for each birth cohort based on the national immunization schedule. The survey design allowed us to assess coverage for vaccines recommended by 12 months of age for all three birth cohorts, for vaccines recommended between 12 and 23 months for two birth cohorts (2013 and 2009), including MMR1, and for vaccines recommended between 60 and 71 months, including MMR2, for the birth cohort of 2009.

Because of very recent introductions, we did not assess coverage for pneumococcal conjugate vaccine for 2014 birth cohort or for Hib vaccine for 2009 birth cohort. Tetanus-diphtheria (Td) vaccine recommended at 14 years was not included in the survey.

It was not practical to conduct a household survey for the purpose of coverage assessment in three age strata. The small average household size (3.3 persons; range, from 2.5 in Racha-Lechkhumi to 4.0 in Achara) (*9*) and small birth cohort in Georgia (approximately 50,000-60,000) would have required selecting a very large sample of households to identify sufficient number of households with children from targeted birth cohorts. The existence of the nationwide Civil Registry database linked to the Immunization Management Module provided an opportunity to conduct the survey targeting individual children rather than households.

Since very few families in Georgia keep their children's immunization cards at home (*8*) and parental recall is not considered a reliable source of a child's immunization history, we

obtained information on immunizations from HCFs where children receive immunization services, in accordance with recently revised WHO guidance on conducting immunization coverage surveys (*10*).

Sampling Frame

The lists of children born in 2014, 2013, and 2009 obtained from the Civil Registry database and linked to the recently introduced electronic Immunization Management Module of the Health Information Management System were used as a sampling frame for the survey. The availability of a highly accurate sampling frame allowed us to include all children in the survey, not only those registered with HCFs on which officially reported administrative coverage data are based.

The Civil Registry database includes information on all children who are born and receive a birth certificate in Georgia. Based on a UNICEF assessment in 2010, the rate of registration at the time of birth was very high (97%) (*11*) and has likely increased since then with further substantial improvement of Civil Registry services. The information available included child's name, date of birth, personal ID number, legal address, and, for a subset of children, the actual address and the name of HCF where the child receives health services. Children living outside Georgia where considered ineligible for the survey. Therefore children with foreign address listed in the Civil Registry database were excluded from the survey (301 [0.5%] children in 2014 cohort, 326 [0.6%] in 2013 cohort, and 497 [0.8%] in 2009), as well as children who were initially sampled but were subsequently found to have moved overseas.

Design and Sample Size

A complex, stratified, multi-stage design was used for the survey (Appendix Table 4). The country was divided into four survey domains consisting of the three largest cities (Tbilisi, Kutaisi, and Batumi) and the rest of the country. In the three large city domains, simple random sampling (SRS) was used to select children [primary sampling units (PSU)] from each of the three age groups. The fourth domain, consisting of the populations not residing in one of the three largest cities, was divided into seven strata. In the first stratum, which included Rustavi and Poti, participants within each age group were selected by SRS because the sampling frame had no easily identifiable subdivisions to be used as sampling units for cluster survey. Five strata required a two-stage cluster design. In the first stage, settlements (village/town) were selected by

probability proportionate to population size (PPS), followed by an SRS of children within each age group. The last stratum, representing the remaining 54 districts of Georgia, required a 3-stage cluster design. In the first stage, districts were selected by PPS, followed by selection of settlements (village/town) by PPS, followed by a SRS of children within each of the three age groups. Very small settlements were pooled to create sampling unit with >10 children in it.

A sample size of 750 per birth cohort was allocated to Tbilisi (representing 3.8% of all children), and 600 per birth cohort to Batumi (20.0%) and Kutaisi (22.1%), resulting in 1950 children per birth cohort for the three cities combined. In the rest of Georgia domain, a sample size of 50 per birth cohort was allocated to Gori and combined Rustavi/Poti stratum. A sample size of 25 per birth cohort was allocated to the next four strata (five per PSU). In the last stratum, a sample size of five children per SSU was allocated, resulting in 25 children per PSU. This resulted in 800 children per birth cohort in the fourth domain (2.4% of all children). In total, 2,750 children per birth cohort were selected, which resulted in a total sample size of 8,250 children for all three birth cohorts included in the survey.

Selection of sampling units was performed using the population data for the 2014. Individual children were selected from the sampled units using line-lists for respective birth cohorts.

Upon survey implementation, of 8,250 children selected in the three birth cohorts, 103 (1.2%) were found to have moved to other countries, resulting in 8,147 children eligible for the survey. We obtained immunization information for 7,723 of them for an overall enrollment rate of 94.5%, and 424 (5.2%) children could not be found. In all birth cohorts and domains, >90% of eligible participants were enrolled (range, 90.4%–98.0%).

Survey Procedures

The relevant population subsets were extracted from the Civil Registry birth registration database via the Immunization Management Module link. The residence codes were assigned to each administrative unit based on child's address. If actual address was different from the child's legal address, the actual address was used to assign the child to sampling unit, accounting for some population movement and reducing the proportion of children who could not be located.

Participant selection process was performed by survey coordinators. SRS was applied using an online random number generator (www.random.org). The survey field teams were given lists of selected children with their addresses and, if known, HCF indicated in the Immunization Management Module (the list and contact information of HCF is available through the Health Information Management System). For children with known HCFs, the teams visited HCFs to locate the immunization records of children selected for the survey.

If the child's immunization records could not be located at the listed HCF or no HCF was listed, the teams visited the child's residence and, after providing an information sheet about the survey, asked parents/guardians if the child had received at least one vaccination. If the answer was positive, parents/guardians were asked to provide information about HCF where the child receives immunizations. If the immunization card was available at home, the data were obtained on-site. Otherwise, the team visited the HCF indicated by a parent/guardian to obtain immunization records. If the child was unvaccinated per parent/guardian report, this information was noted in the interview form and no further attempts to locate records for this child were undertaken. Children who could not be found were not replaced by selecting another child.

The information collected on survey participants included date of birth, sex, residence district/city, HCF, vaccine doses received and dates of vaccination. The information was recorded on a survey data collection form.

To accommodate the timeframes of availability of staff and funding, the survey was implemented sequentially in Batumi in August 2015, in Kutaisi in September 2015, in Tbilisi in March 2016, and in the rest of Georgia in August-October 2016. To reduce the impact of sequential timing of survey implementation, immunization records for the children in Batumi and Kutaisi who had not reached full year of the cohort age at the time of initial field work (were born in the late months of year) and had not received all age-eligible vaccines were reviewed again at HCFs or via Immunization Management Module in early 2016, and any additional doses received were noted.

The survey field teams were comprised of personnel from NCDC, CDC/GID, CDC South Caucasus Office, FELTP graduates and from local Public Health Centers of survey areas. Before beginning fieldwork, the survey personnel received comprehensive training on the survey objectives, methodology, and procedures for data collection.

Data Management and Analysis

The statistical software Epi Info 7 was used for data entry. Analysis was conducted using SAS v9.4 and R v3.3. Analyses accounted for the complex survey design and sampling weights. We report Wilson-Score confidence intervals for proportions using survey procedures in SAS 9.4. Main outcome measures included per cent coverage for MMR1 and MMR2. Overall coverage for MMR1 and MMR2 at the time of the survey and the timely coverage at standard time points – by 24 months for MMR1 and by 72 months for MMR2 were calculated.

To account for differences in the time of observation, comparisons across cohorts were made based on the timely coverage. To remove the impact of the sequential implementation of the survey in different domains on the coverage levels, we calculated coverage for each dose by the time of the end of the initial field work in Batumi (the city surveyed first), by excluding any vaccine doses administered after September 1, 2015. Direct comparisons across survey sites were made based on the status as of September 1, 2015.

The estimates of coverage were compared to the national target of 95% coverage for all doses. The target does not specifically refer to timely coverage, therefore, in the analysis we applied it to overall coverage by the time of the survey. The survey results were also compared to corresponding administrative coverage reported through GEOVAC system. GEOVAC, the existing system for administrative reporting of coverage in Georgia, is based on the data provided by HCFs to NCDC and only reflects children registered with HCFs.

Ethical Issues

The coverage survey protocol was reviewed by Human Subject Research Coordinator, GID/CGH/CDC and Ethical Committee, NCDC, and determined to be an evaluation of public health program rather than human subject research.

References

1. Ministry of Labor. Health and Social Welfare of Georgia. National guidelines for measles, rubella, and congenital rubella syndrome surveillance and outbreak control. Tbilisi, 2017 [in Georgian] [cited 2019 Oct 3]. http://ncdc.ge/Pages/User/News.aspx?ID=71654a22-1b85-4ecf-ade3-fbdf8668113a

2. World Health Organization. Eliminating measles and rubella. Framework for the verification process in the WHO European Region. 2014 [cited 2019 Oct 2].

http://www.euro.who.int/__data/assets/pdf_file/0009/247356/Eliminating-measles-and-rubella-Framework-for-the-verification-process-in-the-WHO-European-Region.pdf?ua=1

3. National Statistics Agency of Georgia. Population data [cited 2020 Apr 1]. https://www.geostat.ge/en/modules/categories/41/population

4. Khetsuriani N, Wannemuehler K, Geleishvili M, Komakhidze T. Final report—immunization coverage survey in Georgia, 2015–2016. Tbilisi (Republic of Georgia): US Centers for Disease Control and Prevention, Ministry of Labor, Health, and Social Affairs of Georgia; 2017, pp.1–58 [cited 2020 Apr 12]. https://www.ncdc.ge/Pages/User/Documents.aspx?ID=1d6e5458-6504-4e5c-88a1-e547b79d5b47&language=en-US

5. Doshi S, Khetsuriani N, Zakhashvili K, Baidoshvili L, Imnadze P, Uzicanin A. Ongoing measles and rubella transmission in Georgia, 2004–05: implications for the national and regional elimination efforts. Int J Epidemiol. 2009;38:182–91. PubMed https://doi.org/10.1093/ije/dyn261

6. World Health Organization. Officially reported immunization coverage series [cited 2017 Jan 28]. http://apps.who.int/immunization_monitoring/globalsummary/coverages?c=GEO

7. State Department of Statistics, National Center for Disease Control; UNICEF. Republic of Georgia Multiple Indicator Cluster Survey, 1999. 2000 [cited 2017 Mar 14]. https://mics-surveys-prod.s3.amazonaws.com/MICS2/Central%20and%20Eastern%20Europe%20and%20the%20Commonwealth%20of%20Independent%20States/Georgia/1999/Final/Georgia%201999%20MICS_English.pdf

8. State Department of Statistics, National Center for Disease Control; and UNICEF. Georgia: monitoring the situation of children and women. Multiple Indicator Cluster Survey 2005 [cited 2017 Mar 14]. https://mics-surveys-prod.s3.amazonaws.com/MICS3/Central%20and%20Eastern%20Europe%20and%20the%20Commonwealth%20of%20Independent%20States/Georgia/2005/Final/Georgia%202005%20MICS_English.pdf

9. National Statistics Office of Georgia (GEOSTAT). 2014 General Population Census—main results, general information [cited 2017 Mar 14]. http://census.ge/files/results/Census_release_ENG.pdf

10. World Health Organization. 2015 update of vaccination coverage survey manual [cited 2017 Mar 14]. http://www.who.int/immunization/monitoring_surveillance/Briefing_note_CSManual.pdf

11. Georgia UNICEF. Birth registration [cited 2017 Mar 14]. http://unicef.ge/10/Birth-registration/34

Appendix Table 1. Population of Georgia by age group, 2013

Age group, y	Population
<1	49,600
1–4	207,400
5–9	216,000
10–14	210,400
15–19	233,200
20–24	281,100
25–29	280,000
30–39	507,000
40–49	490,600
≥50	1,242,000
Total	3,717,300

Appendix Table 2. Main surveillance indicators for measles reviewed for this report (*1,2*)

Indicator	Definition	Target
Rate of discarded cases	Number of discarded cases per 100,000 population	≥2.0/100,000
Rate of laboratory investigation of cases	Number of suspected cases of measles tested divided by the number of all suspected cases excluding cases that have not been tested but were confirmed by epidemiologic link to another laboratory confirmed case or discarded based on epidemiologic link to a case of another disease, expressed as percentages	≥80%
Timeliness of case investigation	Percent of suspected cases with investigation initiated within 48 h of reporting	≥80%

Appendix Table 3. Official country estimates of immunization coverage with measles-containing vaccines reported to WHO, Georgia, 1990–2014*

Year	MCV1 coverage, %	MCV2 coverage, %
1990	99	N/A
1991	81	N/A
1992	16	N/A
1993	61	N/A
1994	63	N/A
1995	61	N/A
1996	88	N/A
1997	95	N/A
1998	90	N/A
1999	97	N/A
2000	97	N/A
2001	100	8
2002	99	40
2003	80	57
2004	86	75
2005	92	87
2006	95	88
2007	97	92
2008	96	87
2009	83	71
2010	94	84
2011	94	77
2012	93	84
2013	97	89
2014	92	87

*MCV, measles-containing vaccine; MCV1, first dose of MCV; MCV2, second dose of MCV; measles vaccine was used until 2004; measles-mumps-rubella vaccine was introduced in 2004. N/A, not applicable. Source: Official country estimates reported to WHO (*5*).

Appendix Table 4. The design of the coverage survey and sample size per birth cohort, Georgia, 2015–2016*

Domain	Strata	PSU	# of PSUs	SSU	# of SSUs per PSU	# of TSUs per SSU	Design	PSU size	Total children
1	Tbilisi (capital city)	Child	750	N/A	N/A	N/A	SRS	1	750
2	Kutaisi (city)	Child	600	N/A	N/A	N/A	SRS	1	600
3	Batumi (city)	Child	600	N/A	N/A	N/A	SRS	1	600
	Three large cities								1,950
4	Rustavi and Poti (cities)	Child	50	N/A	N/A	N/A	SRS	1	50
	Gori (district)	Village	10	Child	5	N/A	2-stage cluster	50	50
	Kobuleti, Marneuli, Zugdidi, and Gardabani (districts)	Village	5	Child	5	N/A	2-stage cluster	25	25 × 4 = 100
	Remaining 54 districts	District	24	Village or town	5	5	3-stage cluster	25	24 × 5 × 5 = 600
	Rest of Georgia								800
	Georgia								2,750

*PSU, primary sampling unit; SSU, secondary sampling unit; TSU, tertiary sampling unit; SRS, simple random sampling; N/A, not applicable. Rustavi and Poti were combined in one unit for sampling purposes.

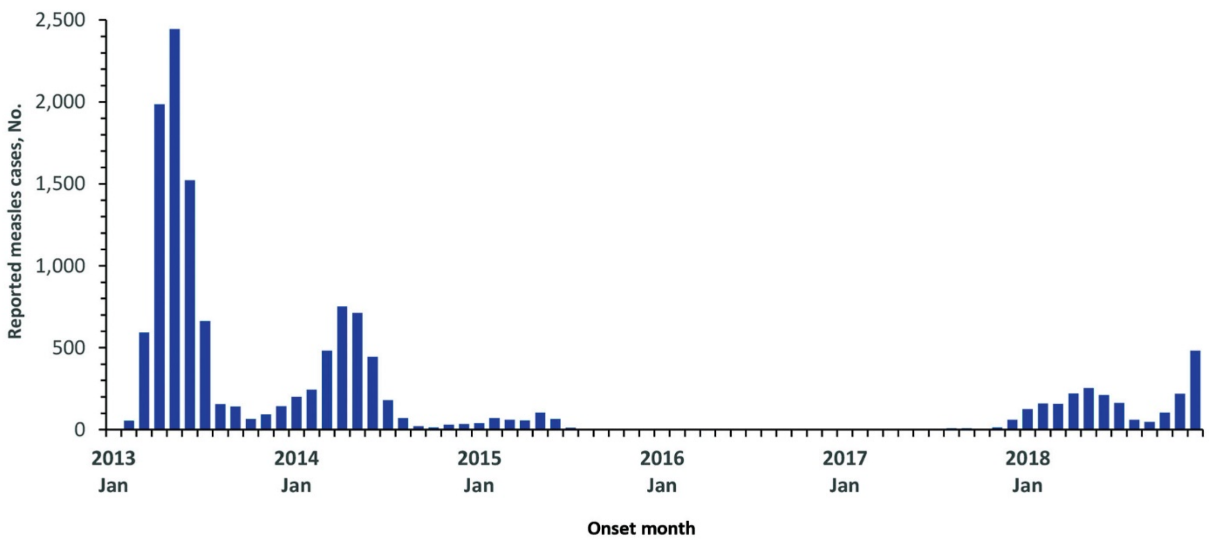

Appendix Figure. Reported cases of measles by month of onset, Georgia, 2013–2018.
